# Outlines of the Diseases of Women

**Published:** 1894-03

**Authors:** 


*Outlines of the Diseases of Women.* By JOHN PHILLIPS, M.D. Pp. xiv., 273. London: CHARLES GRIFFIN & COMPANY, LIMITED. 1893.

This small volume, dedicated to Dr. Playfair, presents us with a good epitome of the modern methods of dealing with diseases peculiar to women, and it may therefore be recommended to the student as serviceable for study, and to the practitioner as useful for reference.

We notice that the author lays it down that "the semen is carried along the Fallopian tubes to the ovary, where the ovum is fertilised, and then carried back along the tubes to the uterus." Is not the uterus alone the seat of normal conception? Is not the function of the ciliated lining of the Fallopian tubes to prevent spermatozoa entering them, and to facilitate the progress of the ovum into its proper nest? It is only in cases where desquamative salpingitis has taken place, destroying the ciliated epithelium, that it would be possible for the spermatozoa to enter the tubes at all.

There are 120 illustrations in this work, most of which are outline drawings by the author. They might be easily copied upon the blackboard by anyone having the most elementary knowledge of drawing. Though they may not all be very artistic, they are useful in rendering clear the meaning of the text.

